# Brief update on coronavirus disease 2019 (COVID-19) diagnostics

**DOI:** 10.1515/almed-2020-0099

**Published:** 2020-11-12

**Authors:** Giuseppe Lippi

**Affiliations:** Section of Clinical Biochemistry, University Hospital of Verona, Piazzale L.A. Scuro, 10, 37134 Verona, Italy

**Keywords:** coronavirus, COVID-19, diagnosis, virus

Coronavirus Disease 2019 (COVID-19), which is caused by a virus called SARS-CoV-2 (severe acute respiratory syndrome coronavirus 2), has now affected several millions of people around the world, causing over one million deaths. SARS-CoV-2 is a typical coronavirus, belonging to the same family of viruses causing flu-like symptoms, as well as those responsible for the two previous outbreaks of severe acute respiratory syndrome (SARS) in China in 2002–2003 and Middle-East respiratory syndrome (MERS) in the Middle-East, in 2012 [1]. Like other members of Coronavirus family, SARS-CoV-2 consists of a single strand, positive RNA (∼30,000 base pairs long), which encodes for both structural and functional proteins. The structural proteins include the so-called spike protein (S), the envelope protein (E), the membrane protein (M) and the nucleocapsid protein (N), which encircles the viral genome. Protein S mediates the bond between the virus and the host cells, thus allowing SARS-CoV-2 to penetrate the host cell and exert its intracellular cytotoxic activity. Among the most important functional proteins, the RNA-dependent RNA polymerase (RpRP) is the enzyme responsible for replication of viral genome within the host cells [1].

Unlike common coronaviruses, which are responsible for upper respiratory tract infections (mostly appearing as cold or flu-like syndromes), SARS-CoV-2 causes a severe pathology, initially affecting the lower respiratory tract (i.e., manifesting with bilateral interstitial pneumonia, with possible progression to acute respiratory distress syndrome; ARDS), but which can then involve many other organs and tissues, ultimately causing the development of a multi-organ failure syndrome (MOF), which may lead to death in over 50% of severe COVID-19 cases [1]. To date, the estimated mortality of COVID-19 is around 3%, therefore nearly 30 times higher than that of influenza (0.1%) [2].

Despite the fact that the biological and epidemiological fate of this disease remains undecipherable and substantially unpredictable, there is common perception that humanity will have to learn to live with SARS-CoV-2, and therefore to manage this new infection, for quite a long time [3]. In this perspective, as also clearly underlined by the mantra “test, test, test” assiduously repeated by the World Health Organization (WHO), the adoption of protocols centred on early, accurate and extensive diagnosis of SARS-CoV-2 infections seems now indispensable, not only for preventing and containing the infection, but also for establishing an appropriate and timely therapeutic management.

Like other infectious diseases, the diagnosis of acute COVID-19 infection is based on detecting the virus or its constituents (especially genetic material and/or proteins) from biological samples. According to the WHO definition, a “case” of COVID-19 is as follows: “*a person with laboratory confirmation of COVID-19 infection, irrespective of clinical signs and symptoms*” [4], so that the number of diagnostic opportunities is broad, differing in terms of target and turnaround time (TAT) [5]. As earlier mentioned, the biological target can be of either genetic or protein nature. According to current WHO guidelines, the identification of genetic material (i.e., RNA) of SARS-CoV-2 represents the reference diagnostic strategy for diagnosing an acute infection [4]. Although saliva appears a valid and even more comfortable alternative [6], nasal and oropharyngeal swabs (collected simultaneously) are still considered the reference material for detecting viral RNA. This diagnostic technique is defined as “molecular”, since is based on amplification of viral nucleic acids (nucleic acid amplification test; NAAT) eventually present in the test sample. The most widespread analytical technique is based on the RT-PCR (reverse-transcription polymerase chain reaction) principle, entails the use of sophisticated instrumentation, is plagued by long TAT (i.e., from 3 to 5 h for obtain test results), and has a modest throughput, since it would only allow to process a relatively modest number of biological samples (usually less than 100, simultaneously). Alongside RT-PCR, other analytical techniques, such as RT-LAMP (reverse-transcription loop-mediated isothermal amplification) are emerging and being increasingly used [4]. These alternative methods, despite characterized by lower diagnostic performance (i.e., lower sensitivity, which translates into reduced efficiency in identifying low viral loads) and being only able to process a limited number of samples at the same time (generally <10), on the other hand will allow to obtain relatively rapid test results (e.g., in less than 45 min) and are therefore suitable for all those circumstances when rapid diagnosis is expressly requested (e.g., for screening symptomatic cases in the emergency room, or testing travellers returning from abroad, after permanence in endemic COVID-19 settings) [7].

**Figure 1:**
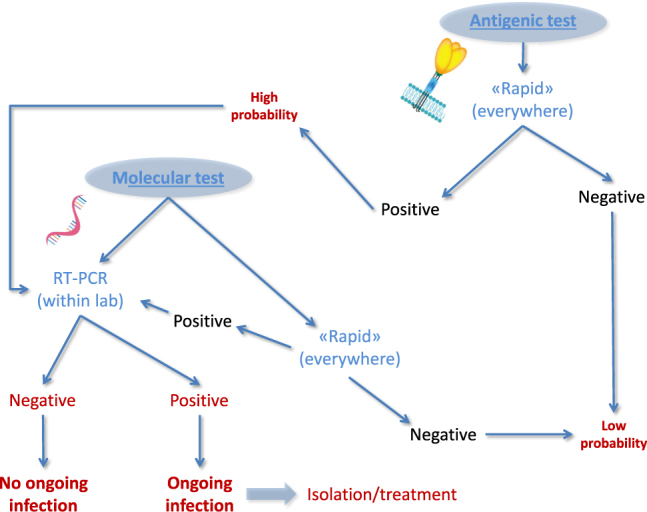
Algorithm for diagnosing severe acute respiratory disease coronavirus 2 (SARS-CoV-2) infection, based on integration of molecular and antigenic testing.

Although the clinical significance of antigenic (rapid) testing is substantially overlapping with that of molecular assays, these techniques deserve a separate discussion. The sensitivity of validated antigen tests (there are now many kits available on the market, some of which fail to display satisfactory diagnostic performance) is reportedly lower compared with molecular testing [7]. In general, rapid antigen tests would allow to identify subjects with medium-to-high SARS-CoV-2 viral load, who incidentally are the most contagious and have a less favourable clinical progression. The tests based on SARS-CoV-2 antigen(s) detection can hence be considered a rapid screening technique, especially in circumstances when many tests would need to be rapidly carried out (e.g., in schools, workplaces, after long-distance trips from high-risk settings such as in airports, railway stations or national borders and so forth). A negative test result could permit to classify the subject as having a “low probability” (of infection and/or infectivity), whilst a positive test result must always be confirmed with RT-PCR, as summarized in Figure 1. Some recent studies have allowed to estimate that the use of antigen tests for screening purposes could allow to reduce the costs of diagnostics by more than 50%, also drastically reducing the time needed for a rule in or rule out [8]. Additional evidence is needed, however, to define the clinical impact of these tests on the secondary attack rate, especially from asymptomatic, pre-symptomatic or mildly symptomatic subjects with low viral load and/or non-viable virus shedding [9].

There is also a third category of diagnostic tests, which falls within the definition of “serology” [4]. This category of tests is designed to detect the presence of a humoral immune response, characterized by development of immunoglobulins (Ig), against the virus, and whose clinical significance is hence not overlapping with detection of viral RNA. This preamble makes it quite clear that the role of serology in acute SARS-CoV-2 infection diagnostics remains very limited, since the appearance of anti-SARS-CoV-2 antibodies is not early (usually occurs 5–7 days after contracting the infection), nor does it allow to distinguishing a recent SARS-CoV-2 contagion from a previous infection [5], [6]. This is almost attributable to the peculiar humoral response developing against this virus, since the appearance of IgM, which usually characterizes the acute phase of most infections, is absent in a variable percentage of COVID-19 patients (from 50 to 90%, especially in those remaining asymptomatic) [10]. It has also been underpinned that IgM appear—at best—concomitantly with, but frequently even later than, IgG and IgA. According to this striking biological evidence, it is hence possible to conclude that serological testing cannot replace molecular and/or antigenic techniques, but will play a definitive role within the context of epidemiological studies and/or health surveillance, for identifying subjects who have been in contact with the virus and have then developed an immune response.
